# Drug checking: a potential solution to the opioid overdose epidemic?

**DOI:** 10.1186/s13011-018-0156-3

**Published:** 2018-05-25

**Authors:** Geoff Bardwell, Thomas Kerr

**Affiliations:** 1 British Columbia Centre on Substance Use, St. Paul’s Hospital, 608-1081 Burrard Street, Vancouver, BC V6Z 1Y6 Canada; 2 Department of Medicine, University of British Columbia, St. Paul’s Hospital, 608-1081 Burrard Street, Vancouver, BC V6Z 1Y6 Canada

**Keywords:** Drug checking technologies, Opioid epidemic, Drug use, Overdose prevention

## Abstract

**Background:**

North America is experiencing an overdose epidemic driven in part by the proliferation of illicitly-manufactured fentanyl and related analogues. In response, communities are scaling up novel overdose prevention interventions. Included are drug checking technologies.

**Main body:**

Drug checking technologies aim to identify the contents of illicit drugs. These technologies vary considerably in terms of cost, accuracy, and usability, and while efforts are now underway to implement drug checking programs for people who inject drugs, there remains a lack of rigorous evaluation of their impacts.

**Conclusion:**

Given the ongoing overdose crisis and the urgent need for effective responses, research on drug checking should be prioritized. However, while such research should be supported, it should be completed before these technologies are widely implemented.

## Background

The opioid overdose epidemic remains the most pressing public health challenge in North America. From 2015 to 2016, overdose mortality rates in the United States increased by 21% to 19.8 deaths per 100,000 individuals, and were as high as 52.0 deaths per 100,000 in the state of West Virginia [1]. In various settings, including the Canadian province of British Columbia (BC), overdose rates have risen due to the proliferation of illicitly-manufactured fentanyl and related analogues. In 2017, an overdose rate of 29.6 deaths per 100,000 was observed in BC – a 42% increase from 2016 [2]. Given the magnitude of this crisis, public health officials have scaled up a variety of overdose response interventions, including supervised consumption sites (SCS) as well as naloxone distribution. Despite such efforts, overdose mortality continues to rise throughout North America, which has prompted the search for novel overdose prevention interventions. Among these are drug-checking technologies (DCT), which are designed to identify the contents of illicit drugs, including the presence of fentanyl and other contaminants. Although little is known about the impact of DCTs in the context of the present opioid overdose epidemic, the excitement about DCTs have prompted some to claim that such technology has “the potential to save hundreds of lives” [3]. In turn, DCTs are now being implemented in settings throughout North America in an effort to address persistently high rates of overdose death [3, 4].

## Drug checking technologies

DCTs range considerably in terms of cost, usability, time required, and data reporting specificities. Initial costs range from $2 (USD) for basic and portable DCTs (e.g., urine test strips) to upwards of $250,000 (USD) for larger and more advanced models (e.g., mass spectrometry). Some DCTs are able to identify common drugs and unknown substances as well as quantify results while others are more limited with regard to specificity and sensitivity, and types of drugs detected [5]. The time required per drug sample ranges from 2 min to hours depending on the DCT, and whether transport of samples is required. These run times for the DCTs do not include collection, preparation, or report generation [5, 6]. At this time, little is known regarding how such differences in technology and process affect uptake and outcomes of DCTs.

Although a small number of studies on the implementation of DCTs within drug user communities have been undertaken, until recently these focused primarily on drug use within dance or other nightlife settings [5] and there is limited rigorous evaluation of their impacts [6]. There are, however, a small number of studies of DCTs use among people who use opioids, including people who inject drugs (PWID). The results of feasibility studies examining expressed willingness to use DCTs among PWID have varied, with willingness ranging across settings from 33 to 90% [7–9]. Feasibility data aside, preliminary findings from an actual fentanyl drug-checking program at a SCS in Vancouver, Canada indicated that a positive fentanyl result was associated with higher odds of dose reduction [10]. However, a concerning finding was that only 1% of SCS clients used this program when it was offered, although the extent to which issues with program design or implementation affected uptake is not known.

DCTs have also been used in several countries for public safety and surveillance purposes [5]. Monitoring illicit drug markets on an ongoing basis could potentially offer some benefit and allow health officials to issue warnings regarding the toxicity of illicit drugs in local markets. However, past qualitative research on drug warnings for PWID has raised questions about the effectiveness of such warnings, as they do not appear to prompt changes in drug use behaviour [11]. However, it may be that the surveillance opportunities afforded by DCTs may extend beyond overdose warnings and include informing outreach strategies, although there currently exists no data to support such applications. A further concern relates to the fact that many DCTs rely on a reference library for detection. Although a recent study found that a few select DCT technologies were able to detect a small number of fentanyl analogues [12], given the ongoing emergence of new fentanyl analogues and other contaminants it remains unclear whether DCT systems will be able to keep up with such developments. A failure to do so could result in an inability to detect a potentially fatal substance in a given sample. All considered, the potential impact of DCTs within the context of the current overdose epidemic is difficult to assess given these limitations.

## Conclusions

Given the lack of rigorous DCT evaluations involving people who use opioids, further research on DCTs should now be prioritized to determine the true impacts of different DCT models across settings. These evaluations should include an examination of the uptake of DCTs by type of drug use (e.g., stimulant vs. opioids); impacts of wait times and differences in technologies on use of DCTs; impacts of DCT results on drug disposal, dose reduction, and other drug use patterns; effects on use of specific supply sources (e.g., dealers) and drug markets; cost/benefit analyses; and potential unintended effects (e.g., failure of detection resulting in overdose). Furthermore, evaluations should also consider different drug use settings (e.g., level of fentanyl saturation of drug markets), how the features of such settings impact use and outcomes of DCT programs, and how different DCT services (e.g., brief counseling) may serve to reach hidden PWID.

To combat the overdose crisis that is affecting communities around the world, government and public health officials must continue exploring, implementing, and evaluating novel overdose response interventions. However, given the lack of rigorous evidence supporting the real-world effectiveness of DCTs, only modest and selective implementation of DCTs accompanied by evaluation of outcomes should be considered at this time. Implementation in the absence of rigorous evaluation could result in the wasting of precious resources, and more importantly, more lives lost to fatal overdose.

## Abbreviations

BC: British Columbia
DCT: drug checking technology
PWID: people who inject drugs
